# An update on the Pharmacovigilance Programme of India

**DOI:** 10.3389/fphar.2015.00194

**Published:** 2015-09-22

**Authors:** Ratan J. Lihite, Mangala Lahkar

**Affiliations:** Department of Pharmacology, ADR Monitoring Centre (Pharmacovigilance Programme of India), Gauhati Medical College and HospitalGuwahati, India

**Keywords:** ADR, Pharmacovigilance, PvPI, AMC

Pharmacovigilance is a pharmacological science related to the detection, assessment, understanding and prevention of adverse effects, particularly long-term and short-term adverse effects of medicines (WHO-Essential Medicines and Health Products, 2002). It has been observed that a medication that is proven efficacious in large patient population often fails to work in some other patients of different ancestry. Ancestral background of the patients are controlled by genetic factors that influence drug response-drug targets, drug-metabolizing enzymes, drug transporters, and genes indirectly affecting drug action can modulate drug toxicity and contribute to its individual variability (Ma and Lu, 2011). Thus, adverse drug reactions are highly variable in individuals and are major limiting factor in drug therapy and development. For example people with Asian ancestry are at greater risk for serious cutaneous reactions when starting treatment with carbamazepine. Therefore, even though the drug had already been approved in some other country, clinical trial with robust pharmacovigilance monitoring is needed in the population of different race & ethnicity.

In India, a formal ADR monitoring system was started in 1986 with 12 regional centers. In 1997, India became the member of WHO Programme for International Drug Monitoring managed by the Uppsala Monitoring Centre (UMC), Sweden. At inception, 6 regional centers were set up in Mumbai, New Delhi, Kolkata, Lucknow, Pondicherry, and Chandigarh for ADR monitoring in the country (Gupta, 2010). Of these 6 centers, only the centers in Mumbai and New Delhi were active and thus spontaneous reporting of ADRs were poor. Therefore, in November 2004, Govt. of India has launched National Pharmacovigilance Programme (NPvP) with an annual grant of US$0.1 million approved for 5 years from World Bank (Gupta, 2010). However, the World Bank funding for this programme was ended in mid-2009 and this programme was temporarily suspended.

Recognizing the need for improved ADR monitoring in the country, in July, 2010, under the aegis of Health Ministry, a nation-wide revised ADR monitoring programme was launched and named as Pharmacovigilance Programme of India (PvPI) (Kalaiselvan et al., 2014a). Initially, under this National programme, All India Institute of Medical Sciences, New Delhi was the National Coordination Centre (NCC) and in April, 2011, it was shifted to Indian Pharmacopoeia Commission (IPC), Ghaziabad. Dr. G. N. Singh, Scientific Director of IPC was designated as a National Coordinator of PvPI for ADR monitoring in the country (Rehan, 2013). Under PvPI, ADRs are being identified and spontaneously reported by the healthcare professional of Adverse Drug Reaction Monitoring Centres (AMC). These AMCs are set up across the country in medical colleges approved by Medical Council of India (MCI) (Rehan, 2013). These AMCs are responsible for collecting adverse event as per Standard Operating Procedure (SOP), performing follow up if require for the completeness of ADR reports and uploading these reports in net-based software used for ADR reporting called as Vigiflow (Kalaiselvan et al., 2014b). These drug safety information/Individual Case Safety Reports (ICSRs) are collected in predesigned suspected ADR reporting form, broadly consist of 4 sections i.e., patient's information, suspected adverse reaction, suspected medication(s), and reporter's information. These ICSRs are then reported to NCC for Quality & Signal Review via Vigiflow after causality assessments of ADRs performed using the WHO-UMC causality assessment system (Figure 1). The purpose of this programme is to collect, collate and analyze this reported data to arrive at an inference to recommend regulatory interventions for safeguarding the health of Indian population by ensuring that benefit outweighs the risks associated with the use of medicines. Under PvPI, AMC plays a vital role in collection and follow-up of ADR reports from healthcare professionals. Initially there were 22 AMCs in the country. At present there are 150 AMCs under this programme and categorized into four zones i.e., North, South, East and West (Pharmacovigilance Programme of India (PvPI) newsletter, 2013)^1^. In coming year, there will be 350 AMCs across the country to make this programme one of the largest Pharmacovigilance Programme in the world.

**Figure 1:**
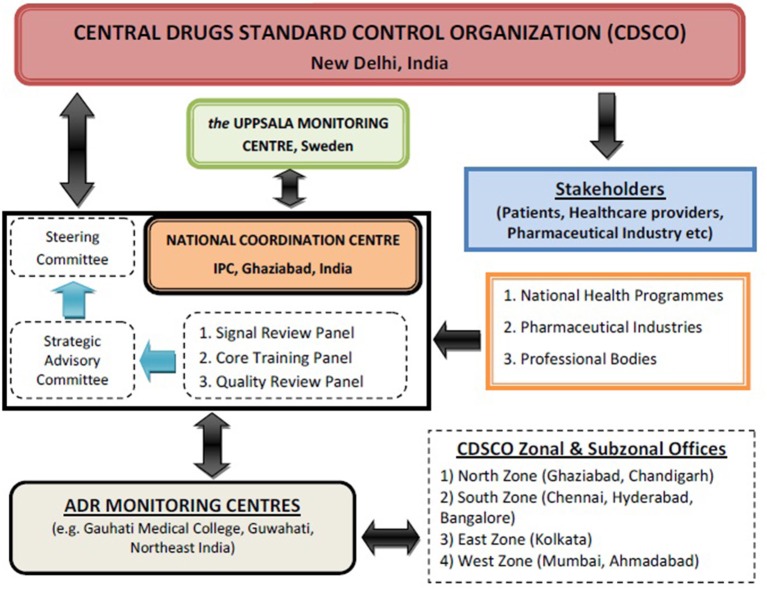
**Programme Communication & flow of ADR reports**.

Under previous National Pharmacovigilance Programme, 11633 ICSRs were reported from January 2006 to December 2008 whereas under PvPI, till June 2014, 78672 ICSRs are reported (Kalaiselvan et al., 2014b). Thus, it can be observed that the rate of reporting has been increased under PvPI. The safety database of PvPI is growing with the increase in number of AMCs in each year. This database allows healthcare providers and consumers to browse and view data on suspected adverse drug reactions of various medicinal products. All data contained herein is sourced from VigiBase®, the WHO global database for ADRs, maintained by the UMC available at http://www.vigiaccess.org. PvPI has generated helpline facility (Tel. No. 18001803024) to make drug safety information available for Indian population. Beside suspected ADR reporting form, PvPI have developed medicine side effect reporting form for consumers/patients in their regional language. PvPI have also extended its reach to other National Health Programmes within country. National coordinating center has collaborated with Revised National Tuberculosis Control Programme and National Aids Control Organization to monitor the safety of drugs use in their programme.

Under PvPI, several drugs are under scanner and quarterly drug safety alerts on suspected unexpected serious adverse reactions (SUSARs) are issued to healthcare professionals via newsletters (Table 1). Based upon PvPI database, this year Drugs Controller of India has instructed manufacturers to include Steven Johnson Syndrome (SJS) in package insert of product containing carbamazepine and advised to the physicians to screen the patients for HLA-B^*^1502 allele before initiating treatment with carbamazepine. However, India doesn't have a strong database on ADRs and has to depend on data from Western countries to take decisions relating to banning and suspension of drugs. The present database of PvPI available on ADRs is not sufficient to represent the population which consumes the drug or to which the drug has been prescribed for. Epidemiological data on drug utility and outcomes of treatments is inadequate. Therefore, for sufficient database on ADRs, awareness among the healthcare providers of government and corporate hospitals including rural areas are needed to be created. The other healthcare institutes like dental, pharmacy, nursing, paramedical etc. associated with patients care by providing safe and effective medication should be encouraged for ADR reporting. Beside these, pharmaceutical companies need to be involved in PvPI for better pharmacovigilance system. Furthermore, incorporating a chapter on pharmacovigilance in education curriculum of medicine, pharmacy, nursing etc. could generate the culture of ADR reporting among young scholars.

**Table 1:** **SUSARs reported during 2011–2013**.

**Sl. No**.	**Drug name**	**Reported ADR**	**SUSARs in PvPI database**	**Global drug safety database (Vigibase)**
1	Sodium valproate	Slurred speech	11	14
2	Streptokinase	Hepatitis	4	18
3	Liraglutide	Ischemic coronary artery disorder (Anginal pain)	2	45
4	Bupivacaine	Confusional state	2	13
5	Omeprazole	Hypokalemia	1	49
6	Furosemide	Breathing abnormalities (Tachypnoea)	1	33
7	Nitrofurantion	Lip oedema	1	9
8	Amphotericin-B	Hypernatremia	1	6
9	Tramadol	Renal & urinary tract neoplasm	1	5
10	Metronidazole	Thrombophlebitis	1	2
11	Gentamicin	Thrombophlebitis	1	1

It was observed that the percentage of ADR reporting by physicians was higher as compare to pharmacists and other healthcare providers (Kalaiselvan et al., 2014a). In India, system of distribution does not leave much scope for pharmacists, nurses, and other healthcare providers to be a significant source of ADR reporting. Even though nurses are in closer contact with the patients for a longer duration, in the event of ADRs observed by them, they have to inform to the treating physician. Similarly, pharmacist's can also promote the development, maintenance, and ongoing evaluation of a programme to reduce the risks of ADRs by detecting, reporting, and assessing any suspected ADRs. Therefore, co-ordination among clinician, pharmacist, and nurse appears vital in contributing each of their respective expertise and experience to promote the rational use of medicines. It was also observed that the lack of knowledge of where, what and how ADRs should be reported is also affects reporting. The reason for poor reporting may also include financial incentives, ignorance (only serious ADRs are to be reported), apprehension of reporting serious ADRs, and lack of time or over load. Thus, healthcare professionals should be under an obligation to report ADR if detected while clinical practice. However, several steps are taken to tackle the problems of under reporting by addressing various issues in various forum and conferences, circulating questionnaire form, writing to professional bodies, scientific journals, etc. In an effort to extent awareness among healthcare providers, continues medical education are being organized in various medical colleges across the country. In addition, Technical Associates are recruited at AMC to facilitate ADR reporting from healthcare providers.

In year 2013, India's contribution to WHO–UMC's global drug safety database (Vigibase) was 2%. India was 7th in position among top 10 counties contributing to global drug safety database. Among Asian countries, India is the only country having more than 1 lakhs ICSRs in Vigibase. According to WHO-UMC Documentation Grading-Completeness Score, the average completeness score of India in 3rd quarter of 2014 was 0.94 out of 1 [(WHO-Uppsala Monitoring Centre (UMC), 2014)]. Thus, from this completeness score it can be predicted that AMCs of PvPI are collecting all the necessary information required for ADR reporting via Vigiflow.

In conclusion, awareness about the ADR reporting among the healthcare providers can improve the rate of reporting across the country. Moreover, by developing own national database and sharing information with other regulatory agencies will provide the much needed information from worldwide data to take the correct decision on medicines and products.

## Conflict of interest statement

The authors declare that the research was conducted in the absence of any commercial or financial relationships that could be construed as a potential conflict of interest.

## Footnotes

^1^http://www.ipc.gov.in/PvPI/pv_about.html.
